# Two excited-state datasets for quantum chemical UV-vis spectra of organic molecules

**DOI:** 10.1038/s41597-023-02408-4

**Published:** 2023-08-21

**Authors:** Massimiliano Lupo Pasini, Kshitij Mehta, Pilsun Yoo, Stephan Irle

**Affiliations:** 1 Oak Ridge National Laboratory, Computational Sciences and Engineering Division, Oak Ridge, 37831 USA; 2 Oak Ridge National Laboratory, Computer Science and Mathematics Division, Oak Ridge, 37831 USA

**Keywords:** Structure elucidation, Computational chemistry

## Abstract

We present two open-source datasets that provide time-dependent density-functional tight-binding (TD-DFTB) electronic excitation spectra of organic molecules. These datasets represent predictions of UV-vis absorption spectra performed on optimized geometries of the molecules in their electronic ground state. The GDB-9-Ex dataset contains a subset of 96,766 organic molecules from the original open-source GDB-9 dataset. The ORNL_AISD-Ex dataset consists of 10,502,904 organic molecules that contain between 5 and 71 non-hydrogen atoms. The data reveals the close correlation between the magnitude of the gaps between the highest occupied molecular orbital (HOMO) and the lowest unoccupied molecular orbital (LUMO), and the excitation energy of the lowest singlet excited state energies quantitatively. The chemical variability of the large number of molecules was examined with a topological fingerprint estimation based on extended-connectivity fingerprints (ECFPs) followed by uniform manifold approximation and projection (UMAP) for dimension reduction. Both datasets were generated using the DFTB+ software on the “Andes” cluster of the Oak Ridge Leadership Computing Facility (OLCF).

## Background & Summary

The ultraviolet-visible (UV-vis) absorption spectrum of an organic molecule interacting with light is a particularly important excited-state property that reveals many of its electronic and optical properties, photochemical reactivity, and chemical reactivity. Applications of photoactive molecules span a wide range of diverse applications, from photovoltaics for solar energy^1^ to electrochromic dyes^2^ for energy-efficient window application, and optical imaging in biological research such as deep-tissue imaging^3^. The discovery of molecules with tailored optoelectronic and photoreactivity properties represents a major challenge for technological advances in these areas. Trial and error-based molecular design is still commonplace but arduous and costly, and it is therefore advantageous to develop computational inverse design capabilities to infer the unknown chemical composition of a molecule matching desirable electronic excitation spectra^4^. Solving this inverse problem within a reasonable time requires an effective exploration of a high-dimensional molecular space characterized by molecules of different sizes and chemical compositions. Quantum chemical electronic structure methods such as multi-reference configuration interaction (MR-CI), complete active space second-order perturbation theory (CASPT2), or time-dependent density-functional theory (TD-DFT), allow to supplant experimental measurements of UV-vis spectra in the gas phase with *in silico* calculations, but the computational time needed to perform these calculations still hampers a rapid exploration of the molecular space^5,6^.

Recent works have shown that deep learning (DL) models can be used as effective surrogates for fast and still accurate estimations of the UV-vis spectra^5–7^. However, a large amount of training data is needed to ensure accuracy, generalizability, and transferability of the trained DL model. In order to collect large volumes of data that can be used to train accurate DL models, high-performance computing (HPC) and permanent data storage facilities need to be leveraged to run quantum chemistry calculations and store large volumes of data^8^.

In response to the need for leveraging large-scale HPC resources for generating large amounts of quantum chemical electronic excitation spectral data, we present two new open-source quantum chemistry datasets called GDB-9-Ex^9^ and ORNL_AISD-Ex^10^ that provide simulated UV-vis absorption spectra for organic molecules. The two datasets differ in the number of molecules considered, as well as in the size of molecules and their chemical composition. These are the largest datasets containing excited states properties of molecules to date. We created them with the goal of providing significant coverage of the chemical and molecular structure space in terms of structural variability, number of atoms contained in the dataset (from 5 to 71 non-hydrogen atoms), and to report statistical analysis for excited state properties in relation to molecular orbital (MO) descriptions. Through the use of the “Atomic Simulation Environment” (ASE)^11^ package, our developed workflow software is agnostic of the quantum chemistry code and thus provides a general capability for generating optical spectra of molecules using higher level electronic structure theories.

## Methods

The simulations for these large datasets of UV-vis absorption spectra were based on the computationally inexpensive density-functional tight-binding (DFTB) method^12–14^ for geometry optimizations of molecules in their electronic ground states, and its excited states extension, the time-dependent DFTB (TD-DFTB) method^15^ for electronic excitation energies and associated oscillator strengths. These semiempirical methods were selected due to the enormous computational cost associated with TD-DFT calculations of such large numbers of compounds. The particular strength of our datasets is the large number of molecular systems they contain, as similar datasets generated with higher level theories contain significantly smaller numbers of molecules^16,17^.

The DFTB method^13,18–20^ is an approximation to density functional theory (DFT), utilizing a minimal basis set in conjunction with a two-center approximation to the electronic Hamiltonian and overlap matrix elements. In short, the DFTB total energy is the sum of an electronic and a repulsive energy contributions, and their calculation requires optimized electronic parameters and diatomic repulsive potential energy functions. When charge transfer or polarization between atoms are explicitly considered, the total DFTB electronic energy E is expressed as a Taylor expansion of the terms of density fluctuations *δρ* around atomic reference densities *ρ*_0_ as^21^ In the DFTB formulation, truncation of this series at various orders is termed as different DFTB “flavors” (DFTB1, DFTB2, etc.) which correspond to various accuracies in the interatomic Coulombic interaction^12–14^. We note that DFTB ground state geometries are typically in excellent agreement with higher level methods such as DFT^13,22^, while absolute transition energies from TD-DFTB calculations are often negatively affected by the minimum basis set methodology^15^. A more accurate variant of TD-DFTB has recently emerged, namely the long-range corrected version of TD-DFTB^23^, but unfortunately the available parameters only span the C, H, N, and O chemical elements^24^, which makes calculations for molecules with S, P, and F chemical elements impossible and would have severely limited the scope of our work. Since the goal of our study is to provide large datasets and the associated workflow software for detailed, statistically meaningful studies of the relationship between molecular structure and optical spectra, we resorted to using the long-established, more traditional TD-DFTB method, as our workflow software is agnostic to the type of electronic structure method employed in the generation of the data. A detailed discussion of the performance of TD-DFTB for excited states energies and spectra was recently reported by Ruger *et al*.^25^.

The simulations of UV-vis spectra in this work were performed as follows. First, the Simplified Molecular-Input Line-Entry system (SMILES) strings of the molecules from the GDB-9 database^26,27^ were converted to a 3D atomic structure and stored in a PDB file after preliminary geometry optimization using the Merck Molecular Force Field (MMFF94) in RDKit^21,28^. The primary information stored in the PDB file archive consists of Cartesian coordinates for each atom in their 3D location in space, along with summary information about the structure, sequence, and experiment. We then performed molecular geometry optimization on the electronic ground state potential energy surface, using the third-order DFTB3 method^20^ in conjunction with the matching 3ob set of electronic parameters and repulsive potentials^29,30^. The empirical *γ*-damping for hydrogen bond correction, and the D3 empirical dispersion correction with Becke-Johnson damping (D3(BJ))^31^ was included to improve the description of noncovalent intramolecular interactions. The DFTB3-D3(BJ)/3ob geometry optimizations where then followed by single-point excited states TD-DFTB calculations based on the DFTB2 method^19^ and the matching mio^19,29,32^ and halorg^33^ parameter sets. For simplicity we only considered singlet excitations. In order to ensure a wide enough coverage of excitation energies even for large molecules, we opted to request the simultaneous calculation of 50 excited singlet states, based on linear response theory using the Casida equation and the ARPACK diagonalizer^34^. The computed singlet excitation energies and associated oscillator strengths can be converted to predict UV-vis absorption spectra^35^, where excitation energies correspond to absorption peak positions, and oscillator strengths provide a good measure of the probability of absorption of visible or UV light in transitions between electronic ground and excited states. All DFTB calculations were performed using the DFTB+ code^36^ (version 21.2) and the wrapper for DFTB+ in the Atomic Simulation Environment (ASE)^11^, which performed an internal conversion of Cartesian coordinates from PDB to the .gen file format.

### Workflow for data generation

The workflow for generating the two datasets is written as a Python program that processes molecules in parallel on a High Performance Computing (HPC) cluster. The gap between the highest occupied molecular orbital (HOMO) and the lowest unoccupied molecular orbital (LUMO), also termed the “HOMO-LUMO” gap, and the excitation spectrum for a molecule is generated from the SMILES string. First, a PDB file is created for the molecule from its SMILES string. The sequence of RDKit operations performed to convert a SMILES representation of the molecule into a PDB file is represented in the following pseudocode.

1. mol = AllChem.MolFromSmiles(smiles)
2. mol = AllChem.AddHs(mol)
3. AllChem.EmbedMolecule(mol)
4. AllChem.MMFFOptimizeMolecule(mol)
5. pdb_block = AllChem.MolToPDBBlock(mol)

DFTB calculations for ground state geometry optimizations followed by calculations of the excited state properties are then run using the PDB data as input. The HOMO-LUMO gap is generated from the output of the DFTB calculations, followed by the calculation of the excitation spectrum.

**Fig. 1:**
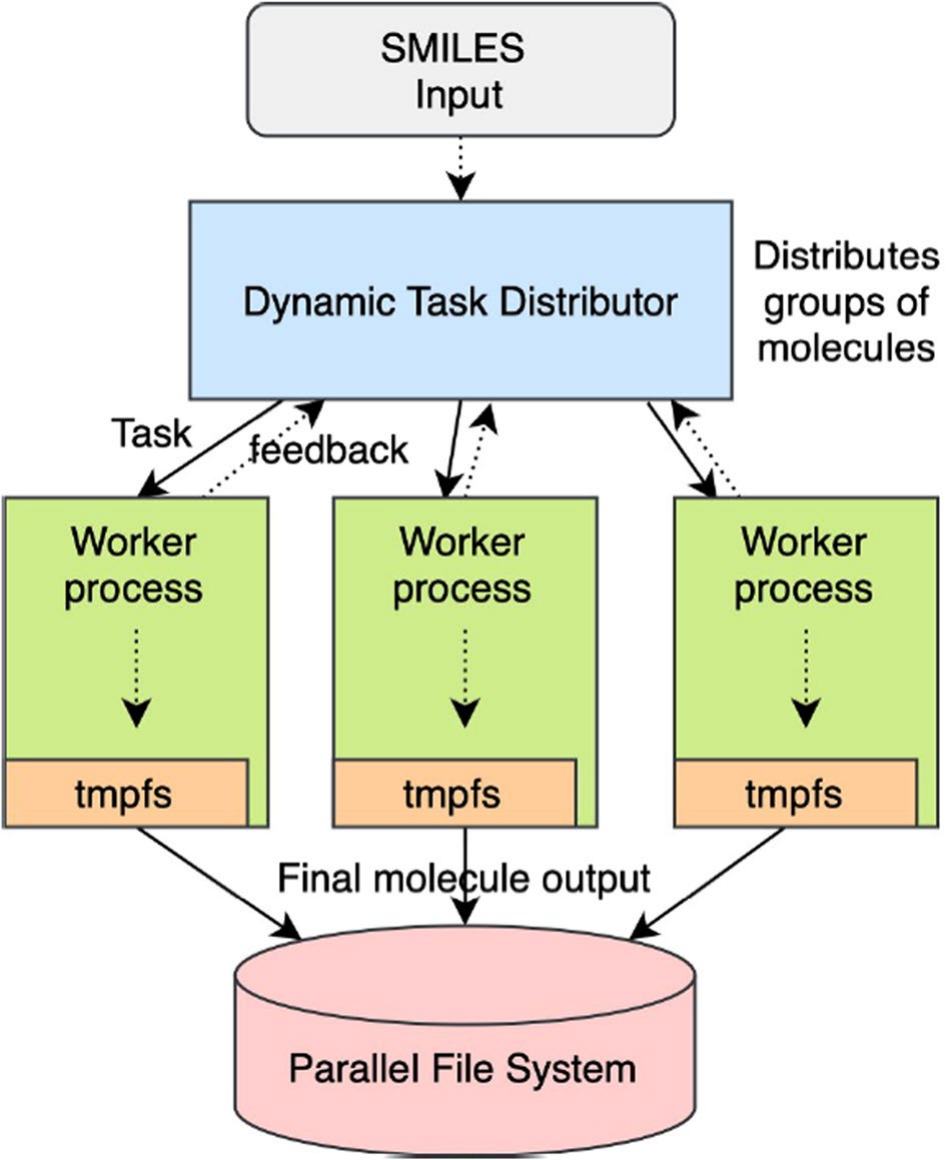
The computational workflow that processes molecules in parallel on a large Linux cluster using a master-worker pattern. The dynamic task distributor helps obtain dynamic load balancing, whereas the file system overhead is mitigated using the hierarchical storage consisting of an in-memory file system and a high-speed parallel file system.

The workflow is run on Andes, a commodity Linux cluster at the Oak Ridge Leadership Computing Facility (OLCF). Molecules are processed in parallel using the Message Passing Interface (MPI), a commonly-used framework for parallelizing scientific applications. As shown in Fig. 1, the workflow uses a master-worker framework in which a co-ordinator process dynamically assigns groups of molecules to worker processes. As the time to process different molecules varies, dynamic task distribution ensures that we obtain efficient load balancing between all worker processes. Each molecule is processed on one CPU core, and the full workflow was run on up to 1,000 cores. When a worker process finishes processing a set of molecules, it requests the co-ordinator for the next set of molecules for processing.

We use an in-memory file system in conjunction with a high-speed parallel file system to efficiently manage over ninety million files generated during the workflow. All output files that include intermediate files created by the workflow for a molecule are first written to the in-memory file system on the compute node. The final set of five files for each molecule is then copied to the parallel file system for persistent storage. Every molecule is assigned a separate directory in which its output files are stored.

Calculating the UV spectrum of a molecule requires performing three main operations:

1. Converting the SMILES string representation of a molecule into a geometric structure where each atom is assigned XYZ coordinates. The geometric structure is written to the file smiles.pdb.
2. Using the file smiles.pdb to compute the relaxed geometry of the molecule, which corresponds with the position of the atoms in equilibrium at the ground state. This generates the files band.out, detailed information about the DFTB run in detailed.out, and the optimized geometry information in the file geo_end.gen.
3. Using the file geo_end.gen to calculate the UV spectrum of the molecule which is written into the file EXC.DAT. Every molecule in the dataset has its own directory.

Note that the default configuration in the *read* function in ASE for reading PDB and optimized geometry data is to have the master MPI process read and broadcast its data to all other processes. To ensure all processes read their own molecule information, this parallel I/O feature was disabled by setting the function argument ‘parallel’ to ‘False’.

After all molecules have been processed, validation codes perform several sanity checks over the entire dataset. Due to the large number of molecules, the validation codes are also developed as parallel programs that run on the analysis cluster at OLCF. For each molecule, they first check for the presence of the five files – (1) the SMILES data in pdb format, (2) the geometry information in the file geo_end.gen, (3) detailed information about the DFTB run in the file detailed.out, (4) band gap information in the file band.out, and (5) the excitation spectrum in the file EXC.DAT. They then perform a correctness check to verify the overall structure of EXC.DAT that contains the UV spectrum. Finally, another parallel workflow generates compressed tar files from the raw data for public release. The list of SMILES strings describing the molecules are obtained from the AISD HOMO-LUMO dataset^37^.

#### Software specification on OLCF andes

The software packages used in this work are installed in a *conda* environment using the popular Conda package management system used in the Python programming ecosystem. In particular, the ASE^11^, DFTB+^36^, and RDKit^28^ packages are installed from the *conda-forge* channel. Table 1 shows the main software components and their versions used for this work.

**Table 1:** Software Specification for the Workflow Components.

Software	Description	Version
ASE^11^	Atomic Simulation Environment	3.22.1
Arpack^34^	Numerical software library	3.7.0
DFTB+^36^	Quantum mechanical simulation	21.2
RDKit^28^	Open-Source Cheminformatics Software	2021.09.5
Python	Programming language	3.9.12
OpenMPI^57^	MPI implementation	4.0.4

### Description of the datasets

Both GDB-9-Ex and ORNL_AISD-Ex datasets contain multiple directories, one for each molecule. The files contained in each molecule directory are as follows: 1. smiles.pdb, 2. geo_end.gen, 3. detailed.out, 4. band.out, 5. EXC.DAT.

To facilitate the consultation of the datasets, we have collected the information of SMILES string, 50 lowest excitation energies and corresponding oscillator strengths in CSV file format. This version of the GDB-9-Ex dataset with compressed information has been released open-source as a stand-alone dataset^38^. We have generated the same compressed version of the data for ORNL_AISD-Ex, which resulted in the generation of 1,000 CSV files. Also this version of the dataset has been released open-source as a stand-alone dataset^39^.

#### Correlation between the HOMO-LUMO gap and the minimum absorption energy

The HOMO-LUMO gap is a quantity that arised from the quasi-particle approximation of the Kohn-Sham formalism^40^. In the exact density functional framework, the energy gap represents the energy required to excite an electron from the ground to its lowest excited state^41^. In many cases, the nature of the first excited state corresponds to a transition of an electron from the HOMO to the LUMO. A previous study on 15 molecules demonstrated a strong correlation between the HOMO-LUMO gap and the minimum excitation energy^42^, and this correlation can be successfully employed in the design of molecular dye molecules^43^. In general, a smaller HOMO-LUMO gap corresponds to a lower minimum absorption energy, indicating that the molecule is more likely to absorb light at longer wavelengths (lower energies). Conversely, a larger HOMO-LUMO gap corresponds to a higher minimum absorption energy, indicating that the molecule is more likely to absorb light at shorter wavelengths (higher energies). However, it is important to note that the correlation between the HOMO-LUMO gap and the minimum absorption energy is not always perfect, as we do not know the exact density functional, and other factors such as different orbital relaxations for HOMO and LUMO orbitals in the excited state can introduce quantitative deviations between the magnitude of the HOMO-LUMO gap and the minimum excitation required to transfer the molecule from ground to first excited state. Factors influencing the overall UV-vis absorption spectrum of a molecule include the *π*-bond conjugation length and aromaticity, steric and ring strain, and clearly the presence of functional groups^4^. It should further be noted that in exact DFT, the HOMO energy is an approximation to the ionization potential (IP) whereas the LUMO energy is an approximation to the electron affinity (EA), as derived from Janak’s theorem^44^. Therefore, the HOMO-LUMO energy gap should be viewed as a proxy for the electrical gap (IP-EA) rather than the optical gap, which differs from the former by the exciton binding energy^45^.

#### GDB-9-Ex

The SMILES strings of the molecules were obtained from the GDB-9 database^26^. The conversion of SMILES strings to 3D Cartesian coordinates of fully DFTB-optimized molecules was successful for 96,766 molecules, for which both geometry optimizations and excited states calculations were successful.

**Fig. 2:**
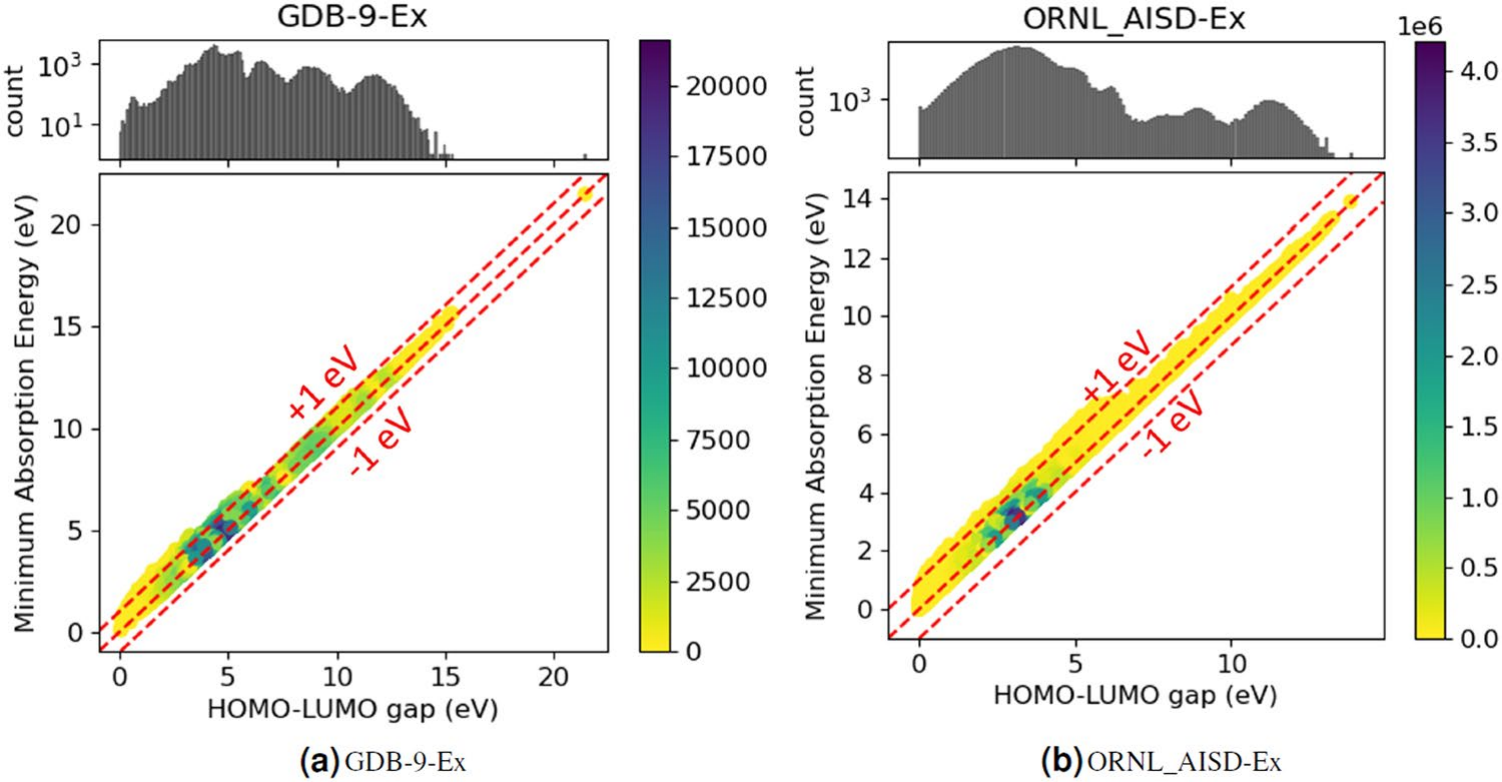
Top: Semi-logarithmic histogram of the HOMO-LUMO gap value across the dataset. Bottom: Parity plot of HOMO-LUMO gap versus minimum absorption energy.

Figure 2 describes the correlation between the HOMO-LUMO gap and the minimum absorption energy for the organic molecules of GDB-9-Ex, confirming the strong correlation between the two quantities. While it is common knowledge that this correlation exists^42^, it has never before been demonstrated to hold on such a large selection of organic molecules. We note that most excitation energies are slightly larger than the HOMO-LUMO gap, indicating that the orbital relaxations in the excited state affect the magnitude of the excitation energies quite systematically. We surmise that this observation could potentially be exploited for data-informed, physics-based predictions of minimum excitation energies from HOMO-LUMO gaps. Interestingly, the illustration shows a single molecule clearly separated from the rest of the molecular dataset, with an HOMO-LUMO gap and minimum absorption energy estimated by DFTB over 20 eV. This molecules is tetrafluoromethane, CF_4_, and the correct estimate of its HOMO-LUMO gap is 15.5 eV according to^46^. Since DFTB and TD-DFTB are minimum basis set methods, they clearly fail to describe accurately the only possible excited state this molecule can attain, the so-called Rydberg excited state^47^, which can be thought of as the transition of an electron from its valence HOMO to the large, diffuse LUMO which is composed of empty unoccupied atomic orbitals, in this case the 3 s and 3p orbitals of C and F, respectively.

**Fig. 3:**
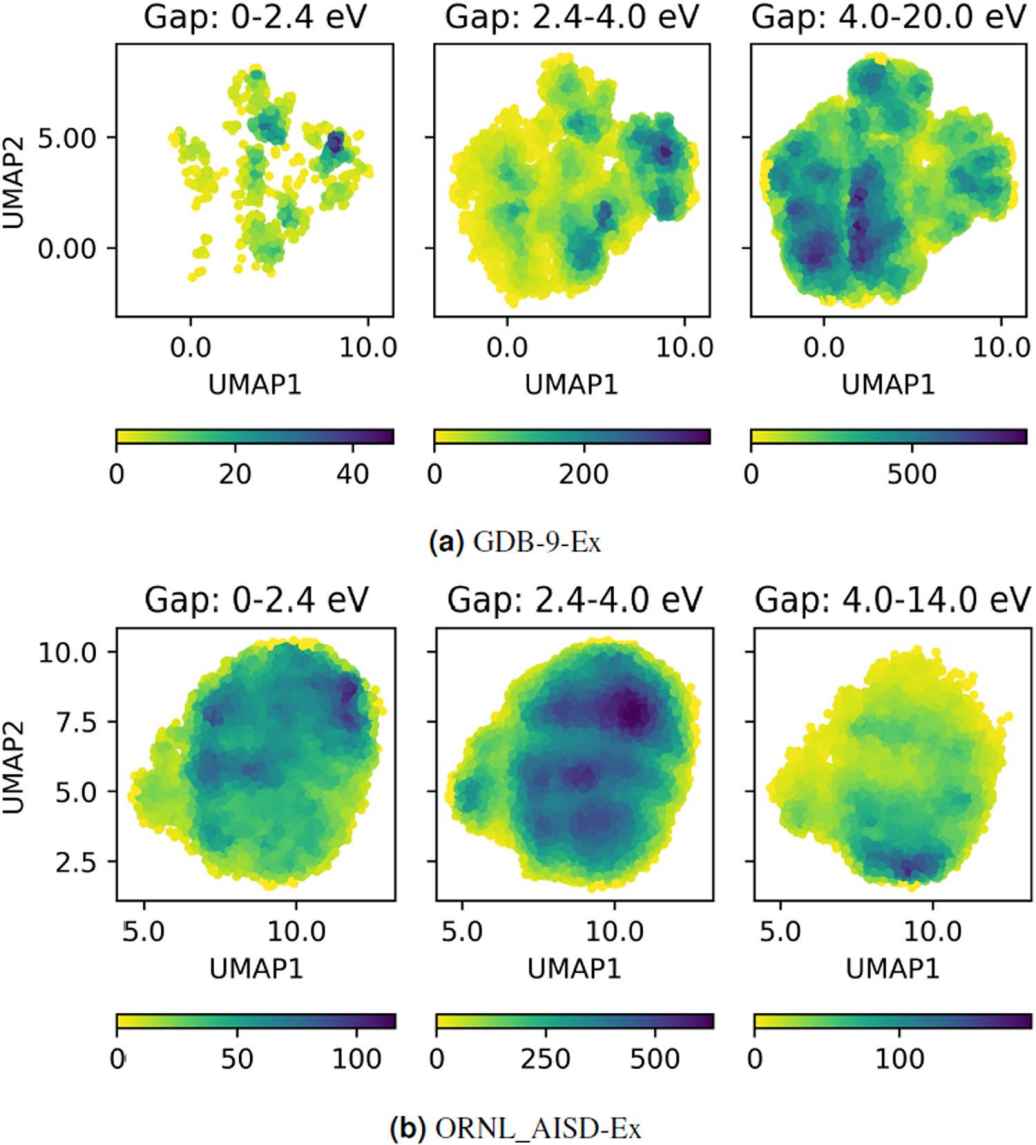
Two dimensional chemical space plot using ECFPs and UMAP dimension reduction for the set of molecules in three ranges (0–2.4 eV (left panel), 2.4–4.0 eV (middle panel) and 4.0–20 eV (right panel)) of the HOMO-LUMO gap. (**a**) the molecule distribution in structural space with all molecules in GDB-9 and (**b**) with 1% of molecules in ORNL-AISD. The color indicates the number of molecules populated in each region of the space.

**Fig. 4:**
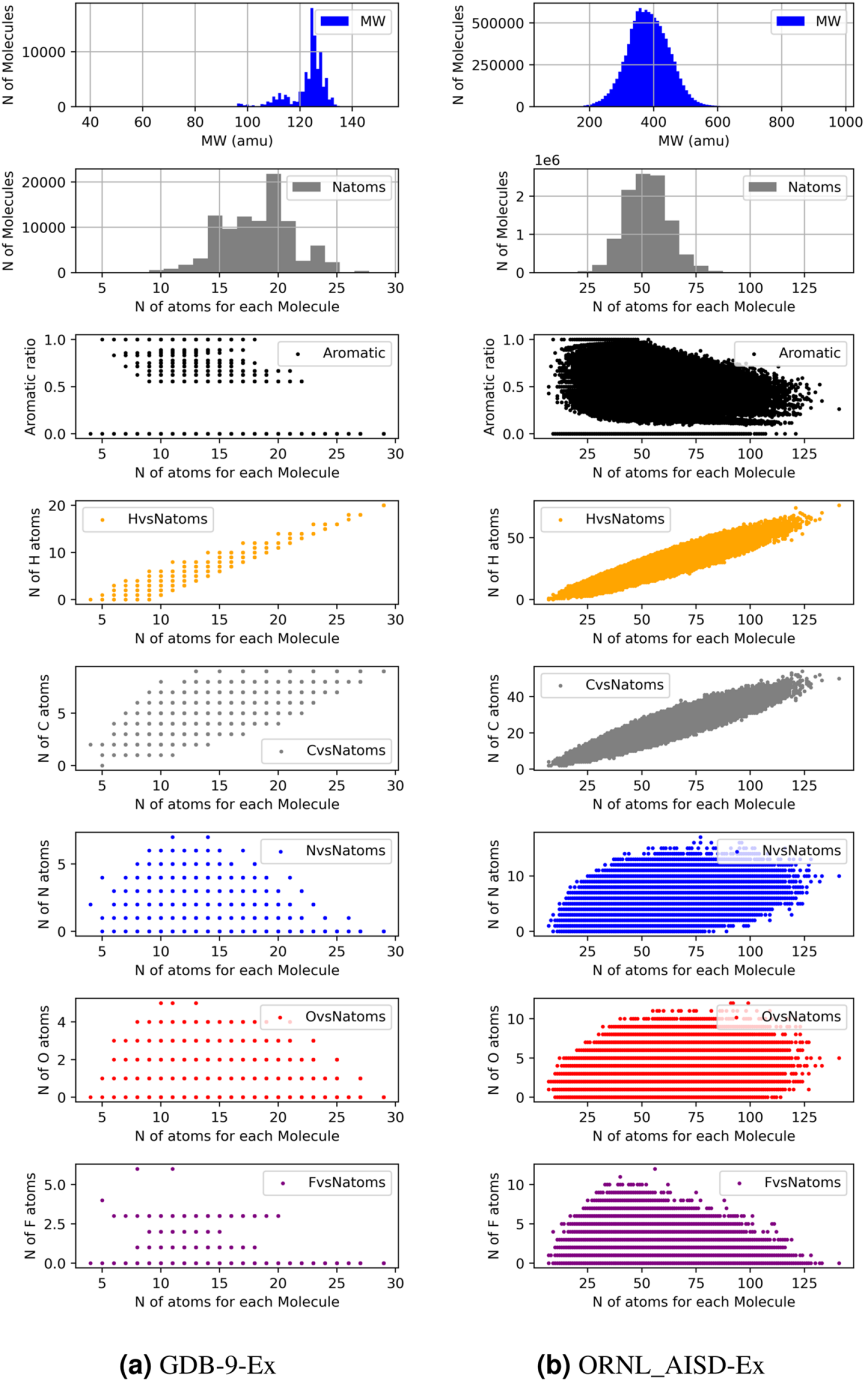
Molecular property analysis for (**a**) GDB-9-Ex and (**b**) ORNL_AISD-Ex. Molecules in both dataset were analyzed with the following properties: distribution of molecular weight (MW), the number of atoms for molecules, the aromaticity ratio and the number of individual elements (H,C,N,O,F) versus the number of atoms per molecule.

Chemical variability of the large number of molecules was examined with a topological fingerprint estimation based on extended-connectivity fingerprints (ECFPs)^48^ followed by uniform manifold approximation and projection (UMAP)^49^ for dimension reduction. Figure 3a shows the distribution of molecules based on the ECFPs and UMAP in three ranges of the HOMO-LUMO gap: the gap of 958 molecules is low, between 0–2.4 eV, the gap of 15,665 molecules is medium with 2.4–4.0 eV, and the gap of 79,112 molecules is high with 4.0–20.0 eV, respectively. These three ranges correspond roughly to the classifications of conductor, semiconductor, and insulator in materials sciences. UMAP dimension reduction was conducted at once for all molecules to consistently compare their relevant position in the chemical space. We note similar features in the UMAPs of low and medium-gap molecules, with very different variability for the high-gap molecules. In addition to the UMAP analysis, we examine the molecular properties such as the number of atoms per molecule, the molecular weight (MW) distribution, the aromaticity (ratio of aromatic atoms to the total number of atoms for each molecule) and the amount of individual element (H,C,N,O,F) of each molecule in Fig. 4 to provide chemical properties of the datasets. Further analysis will be carried out in the future on the molecular structure factors influencing the HOMO-LUMO gap.

**Fig. 5:**
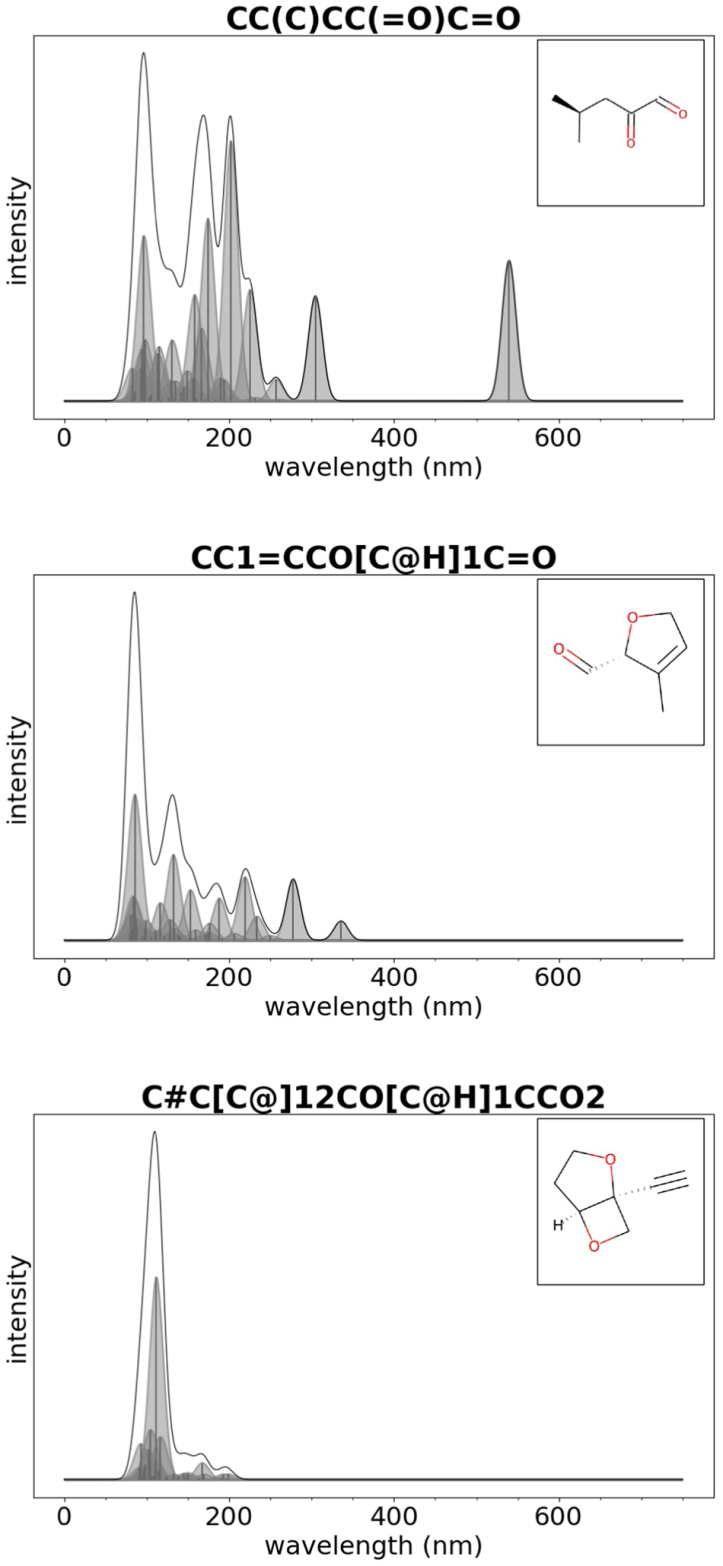
GDB-9-Ex: examples of absorption spectra for organic molecules with HOMO-LUMO gap within the range 0–2.4 eV (top), 2.4–4.0 eV (center), 4.0–20.0 eV (bottom). The title of each figure provides the SMILES representation of the molecule.

Examples of absorption spectra for organic molecules with HOMO-LUMO gap within the range 0–2.4, 2.4–4.0 eV, and 4.0–20.0 eV are shown in Fig. 5. These plots were generated with the Python script dftb-uv_2d.py as explained below.

#### ORNL_AISD-Ex

The molecular structures that we used for ORNL_AISD-Ex were already published in a previous open-source dataset called AISD HOMO-LIMO^37^. These molecules are a subset of a larger dataset generated for previous work^50^, which augmented the Enamine REAL database https://enamine.net/. We refer the reviewer to these publications to obtain more details about how these molecular structures were generated. After preliminary geometry optimization, the SMILES strings of the molecules from the AISD HOMO-LUMO database were converted to a 3D atomistic structure and stored in a PDB file. We note that, since RDKit employs a random choice for the generation of molecular conformers, the molecular geometries obtained in this dataset could be different from the ones obtained when the AISD HOMO-LUMO dataset was generated. The conversion of SMILES strings to 3D Cartesian coordinates of fully DFTB-optimized molecules was successful for 10,502,904 out of 10,502,917 molecules. For these molecules, both geometry optimizations and excited states calculations were successful. The molecules are diverse for chemical compositions (which span five non-hydrogen chemical elements: oxygen, carbon, nitrogen, fluorine, and sulfur) and molecular size (the smallest molecule contains five non-hydrogen atoms, and the largest molecule contains 71 non-hydrogen atoms). The DFTB calculations did not complete for thirteen molecules of the original AISD HOMO-LUMO dataset. We still provide information about the geometry of these molecules. The molecular structures of the thirteen exceptions are stored in a separate tar file named “ornl_aisd_ex_unprocessed.tar.gz” to allow the users to extract information about only these molecules, without necessarily manipulating the whole dataset.

Figure 2b describes the correlation between the HOMO-LUMO gap and the minimum absorption energy for the organic molecules of ORNL_AISD-Ex, confirming the strong correlation between the two quantities. Figure 3b demonstrates the chemical space distribution of molecules in ORNL_AISD-Ex with the ECFPs and UMAP in three range of the HOMO-LUMO gap. The molecules in Fig. 3b were randomly selected by 1% of entire data due to high computation cost. The numbers are corresponding to 11,774 (from 1,177,422) molecules in 0–2.4 eV, 83,488 (from 8,348,848) molecules in 2.4–4.0 eV and 9,752 (from 975,254) molecules in 4.0–14.0 eV, respectively. Both GDB-9 and ORNL_AISD-Ex data sets show similar HOMO-LUMO gap/minimium excitation energies correlations and bear resemblance also in their UMAP dimension reductions, indicating their common molecular origin, albeit with much larger molecular structures present in the latter dataset.

**Fig. 6:**
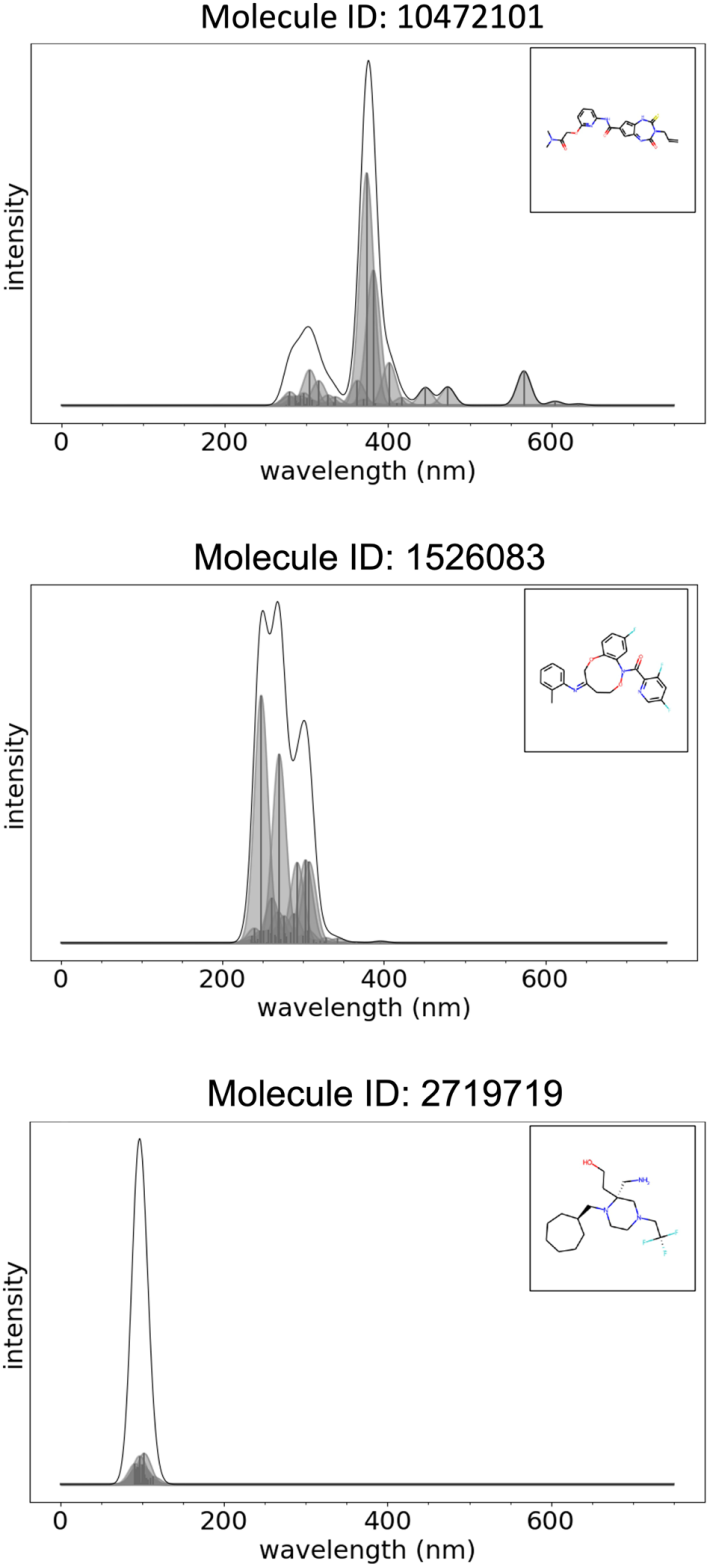
ORNL_AISD-Ex: examples of absorption spectra for organic molecules with HOMO-LUMO gap within the range 0–2.4 eV (top), 2.4–4.0 eV (center), 4.0–20.0 eV (bottom). The title of each figure provide the molecule ID corresponding to the numbering of the molecule in the dataset.

Also for this dataset, we provide examples of absorption spectra for organic molecules with HOMO-LUMO gap within the range 0–2.4, 2.4–4.0 eV, and 4.0–20.0 eV that are shown in Fig. 6. These plots were generated with the Python script dftb-uv_2d.py as explained below.

#### Artefact description

The GDB-9-Ex dataset contains 96,766 directories - one for each molecule in the dataset. However, owing to the large number of molecules in the ORNL_AISD-Ex dataset, its molecule directories are grouped into compressed tar files as explained below.

The ORNL_AISD-Ex dataset consists of 1001 compressed tar files containing a total of 10,502,917 molecules. The tar.gz files are named “ornl_aisd_ex_*n*.tar.gz” where *n* is a numeric value ranging from 1 to 1000. An additional file “ornl_aisd_ex_unprocessed.tar.gz” contains the molecules for which the DFTB calculations could not be completed.

Each tar file contains 10,500 molecules, except for the tar files numbered 34, 121, 128, 352, 360, 429, 495, 509, 518, 627, 676, 668, and 862 that contain 10,499 molecules each. The 13 molecules missing from these tar files could not be processed successfully and are instead recorded in “ornl_aisd_ex_unprocessed.tar.gz”. The last tar file numbered 1,000 contains the remaining 13,417 molecules. The total size of the compressed tar dataset is approximately 75 Gigabytes whereas that of the uncompressed dataset is over 283 Gigabytes.

The molecules in the tar files are ordered according to their position in the CSV file containing the SMILES strings^37^. That is, molecules numbered 0 thru 10,502,917 in the dataset correspond to rows 1 through 10,502,918 in the CSV file. We note that due to array index notation, the molecules in the dataset are numbered starting from 0 instead of 1. The tar file numbering also follows a similar ordering: the first tar file contains the first 10,500 molecules; the second tar file includes the following 10,500 molecules, and so on. This ordering can be helpful for retrieving information about a desired molecule directly. For example, molecule number 1346075 can be found in tar file numbered ┌1346075/10500┐ = 129. The molecule directories for the GDB-9-Ex dataset following a similar numbering notation.

## Data Records

The open-source datasets GDB-9-Ex^9^ and ORNL_AISD-Ex^10^ are stored by the OLCF Data Constellation Facility. The datasets can be downloaded using the Globus data transfer service, as indicated by the instructions provided at the following website https://docs.olcf.ornl.gov/data/index.html#data-transferring-data.

## Technical Validation

The accuracy of the semi-empirical TD-DFTB method for the prediction of UV-Vis absoroption spectra of organic molecules has been evaluated previously on a number of occassions, e.g. against theoretical and experimental best estimates of typical, small molecules^51^, or more recently in a comparison against TD-DFT methods for larger molecules such as rhodopsins and light-havesting complexes^52^. It is clear that the minimum basis set approach in TD-DFTB does not allow the accurate description of energetically high-lying Rydberg states, since unoccupied atomic orbitals such as the 2 s orbital for hydrogen are absent^24^. The minimum basis set also affects negatively the prediction of the oscillator strength and absorption intensities^52^. Nevertheless, agreement of TD-DFTB excitation energies and qualitative features of calculated UV-vis spectra was found satisfactory for organic molecules in many cases^51,52^. At the same time, since TD-DFTB is an approximation to TD-DFT methods, the strengths and weaknesses of the latter matter are inherently present as well, with underestimation of charge-transfer (CT) excited states being one of the most prominent deficiencies^53^. Hybrid functionals such as the PBE0 exchange correlation potential^54^ are able to address this problem in an empirical manner^54^. The most accurate singlet excitation energies for closed-shell organic molecules can be obtained by using *ab initio* correlated electronic structure methods, such as equation-of-motion coupled cluster with single and double excitations (EOM-CCSD), which are completely free from underestimation of CT excitations, but are an order of magnitude more costly than even the TD-DFT methods. For a more extensive discussion on the computational validation of the accuracy attained by TD-DFTB methods in comparison with more accurate (but also more expensive) TD-DFT and EOM-CCSD methods to predict UV-vis spectra, we refer the reader to refs. ^51,52^.

**Fig. 7:**
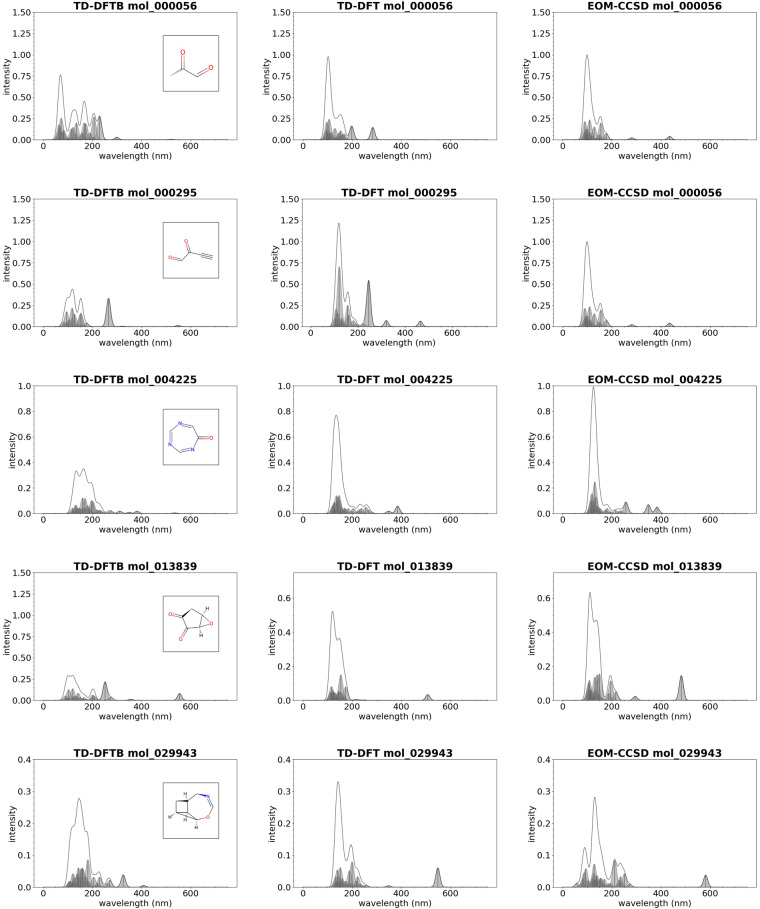
Examples of molecules from the GDB-9-Ex dataset whose UV-vis spectrum has been computed with TD-DFTB (left), TD-DFT with PBE0 as exchange correlation potential (center), and EOM-CCSD (right).

**Fig. 8:**
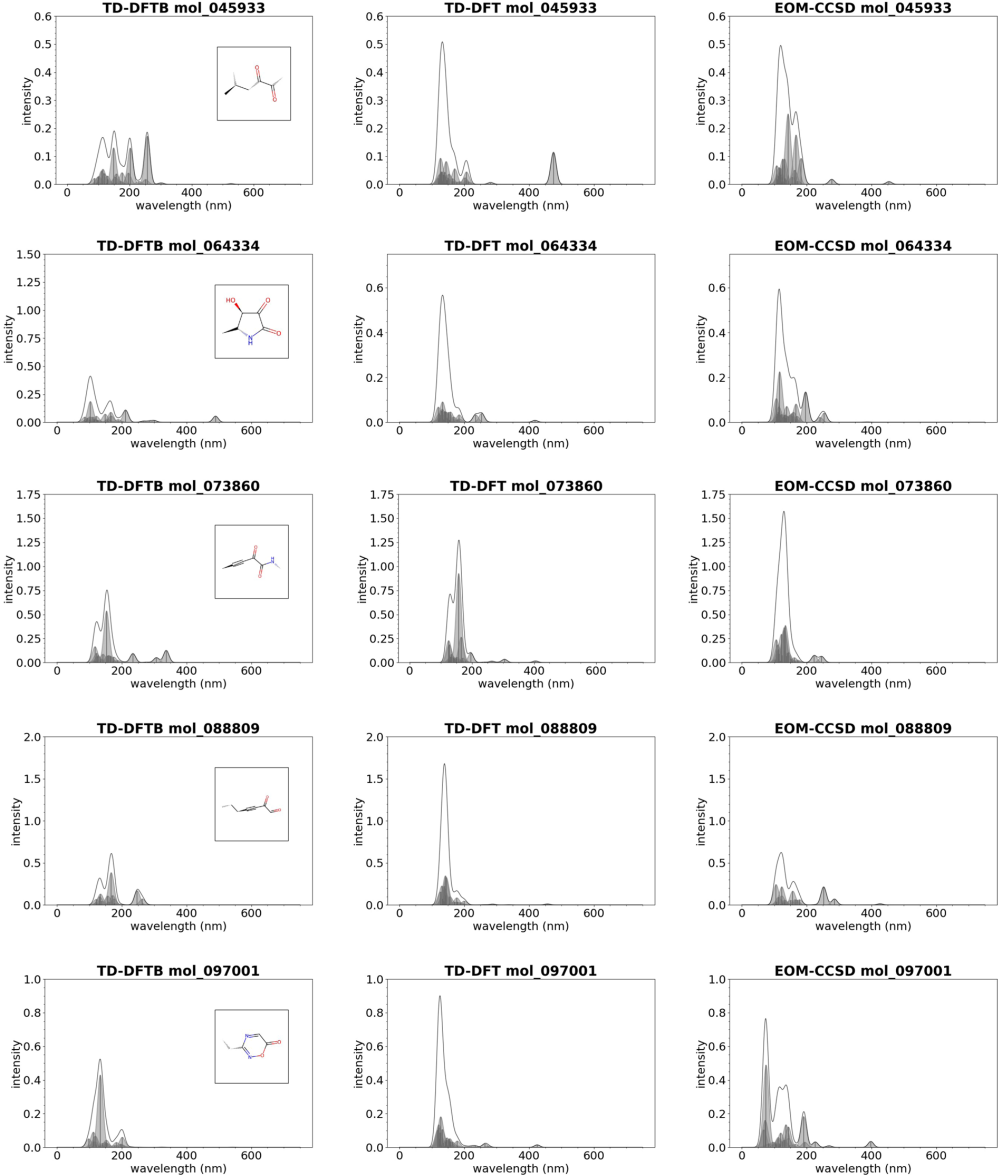
Examples of molecules from the GDB-9-Ex dataset whose UV-vis spectrum has been computed with TD-DFTB (left), TD-DFT with PBE0 as exchange correlation potential (center), and EOM-CCSD (right).

Due to the aforementioned, method-specific shortcomings in the prediction of UV-vis spectra of organic molecules, we resorted in this study to employ two representative methods for validation of TD-DFTB spectra, namely TD-DFT and EOM-CCSD. These calculations have been performed using the ORCA quantum chemistry program package^55^ on a subset of several thousand molecules. We here visually compared 10 molecules that represent a reasonable selection of molecular structure in terms of molecular size, composition, bond structure, “exoticity” (in terms of molecular structure), and different agreements between the three approximation theories. All the molecules selected have intensities between 350 and 750 nm, and the plots of the UV-vis spectrum for these molecules are provided in Figs. 7, 8. We find qualitative agreement between TD-DFTB and both TD-DFT as well as EOM-CCSD methods, while in other cases TD-DFT and EOM-CCSD methods deviate from each other to a similar extent as TD-DFTB from TD-DFT. A systematic comparison of the method capabilities for the prediction of UV-vis spectra for organic molecules is out of the scope of this study, which is focused on the computational workflow to generate UV-vis spectra with arbitrary electronic structure methods and computational codes. We refer the reader to a recent review article related to these topics which covers a broader range of topics related to the selection of the best electronic structure method for the prediction of UV-vis spectra for a specific application^5^.

## Usage Notes

The code for calculating the electronic excitation energies and statistical analysis of the dataset is open-source and available at the ORNL-GitHub repository https://github.com/ORNL/Analysis-of-Large-Scale-Molecular-Datasets-with-Python.

The code contains the following Python scripts:

- xyz2mol.py. Provides a Python implementation of the universal structure conversion method for organic molecules, which creates the three-dimensional geometry from the atomic connectivity as described in^56^.
- mol_remaining.py. Iterates over the dataset, identifies molecules for which the DFTB calculations did not succeed, and writes the ID of these molecules on a text file named mol_remaining.txt.
- smiles_dftb_excited_state.py. The entry point for the main workflow. It implements the master-worker pattern which runs a static DFTB+ calculation to compute the optimized geometry and the HOMO-LUMO gap followed by a time-dependent DFTB+ calculation to compute the UV-vis spectrum for each SMILES string representation of a molecule contained in the.CSV file of the AISD HOMO-LUMO dataset.
- select_molecules.py. Selects molecules based on given criterion and copies them in a new directory.
- dftb-uv_2d.py. Script to collect and plot UV-Vis spectra on both nm and eV scales. Iterates over all the directories associated with each molecule and computes the smoothed spectrum for each molecule, on both nm and eV scales, saving it into the file named EXC-smooth.DAT. The full-width at half-maximum (FWHM) can be arbitrarily tuned by the user with defaults set to 10 nm and 0.5 eV. Total spectral envelopes as well smoothened individual peak contributions and line spectra indicating the calculated excitation energies with associated oscillator strengths as measure for intensity are plotted as well. The Python script supports MPI directives to allow multiple processes to concurrently compute the smoothed spectrum on different molecules. This script is an adaptation of the python script provided at the GitHub repository https://github.com/radi0sus/orca_uv/.
- plot_homo-lumo_vs_minimum_absorption_energy.py. Generates two plots. The first plot shows the correlation between the HOMO-LUMO gap and the minimum absorption energy, which is saved in an image file named HOMO-LUMO_versus_minimum_absorption_energy.jpg. The second plot shows the peaks of the UV-vis spectrum computed with TD-DFTB+ along with the smoothed spectrum, which is saved in an image file named absorption_spectrum.jpg.
- utils.py. Provides basic utilities used by the other Python scripts.

## Data Availability

The code for calculating the electronic excitation energies and statistical analysis of the dataset is open-source and available at the ORNL-GitHub repository https://github.com/ORNL/Analysis-of-Large-Scale-Molecular-Datasets-with-Python.
